# Radiomics based on artificial intelligence in liver diseases: where are we?

**DOI:** 10.1093/gastro/goaa011

**Published:** 2020-04-07

**Authors:** Wenmo Hu, Huayu Yang, Haifeng Xu, Yilei Mao

**Affiliations:** Department of Liver Surgery, Peking Union Medical College Hospital, PUMC, Chinese Academy of Medical Sciences, Beijing, P. R. China

**Keywords:** artificial intelligence, radiomics, radiogenomics, hepatocellular carcinoma, non-alcoholic fatty liver disease

## Abstract

Radiomics uses computers to extract a large amount of information from different types of images, form various quantifiable features, and select relevant features using artificial-intelligence algorithms to build models, in order to predict the outcomes of clinical problems (such as diagnosis, treatment, prognosis, etc.). The study of liver diseases by radiomics will contribute to early diagnosis and treatment of liver diseases and improve survival and cure rates of liver diseases. This field is currently in the ascendant and may have great development in the future. Therefore, we summarize the progress of current research in this article and then point out the related deficiencies and the direction of future research.

## Introduction

Due to the rapid development in the field of computer science in the last several decades, especially in artificial intelligence (AI), concepts like machine learning, deep learning, and big data have been applied in many aspects. As for medical imaging, since judgment and diagnoses made by radiologists are usually the combination of multiple years of training, working experience, and individual interpretation, the results of the same medical image can be subjective and variable among different experts. Also, a large scale of information that cannot be exploited by the human eye resides in medical imaging, which may contribute to the correctness of diagnosis. Taking a mass lesion in a liver as an example, though a list of phenomena such as its shape, density, time, and degree of contrast enhancement has been summarized to distinguish between benign and malignant lesions, many cases belonging to the gray area are always difficult to judge for sure. Therefore, it raises the question of whether the techniques of AI can be taken advantage of to utilize information in medical imaging to a larger extent and thus help us to make more objective and accurate clinical decisions.

## Terminology and general approach

In 2012, Lambin *et al.* [1] proposed the approach of radiomics, which refers to high-throughput mining of data from medical imaging in order to improve the accuracy of clinical decision-making. Usually, a radiomics study can be divided into five steps:

- First, based on the prediction target such as diagnosis, prognosis, therapeutic selection, or gene expression, investigators choose the appropriate imaging protocol like CT, MRI, ultrasonography, etc.
- Second, the region of interest (ROI), an area from which the data are extracted, should be designated either manually by physicians or automatically achieved by algorithms. The ROI can contain the region of tumors only, or with tumors’ habitats, or even include the whole area of the organ, decided by the type of question that is of interest.
- Then, quantitative features are extracted from the ROI by different algorithms. For instance, texture analysis is a commonly used method to extract features that depict the texture information of an ROI by quantifying the distribution of the gray level of every pixel. It can be further divided into first- and second-order texture analysis to describe the intensity distribution only or the spatial relationship of the different gray levels respectively [2]. Obviously, there are other strategies to extract different aspects of features, which is not the key point of this review.
- Next, the extracted features are then connected to the clinical information to see whether they are correlated or irrelevant. Features that are highly correlated may have the potential to predict clinical outcomes and will be involved in building the prediction model. Different machine-learning algorithms, including logistic regression, LASSO Cox regression, random forest, etc., are brought into the process of feature selection. Notably, deep-learning methods such as neural networks do not extract features, but feed the networks with primitive data. Even though it is difficult to understand the way in which deep learning works, as there is no clear feature in constructing the model and the practical effect awaits more research to testify, it is a more promising method by simulating the feedback mechanism like neurons do in the human brain.
- Finally, the prediction model will be validated and its performance improved by further data sets. Radiomics can analyse every tiny part of the medical images, including both the ROI such as the tumor sites and the surrounding tissue. Also, the medical images can be done multiple times with little harm at any point to longitudinally monitor the course of the disease or recovery. Thus, compared with conventional methods like biopsy, radiomics is very suitable for investigating oncology, since tumors have high spatial and timing heterogeneity [3]. Nowadays, much of the radiomics research has focused on the cancer field, such as lung, colorectum, brain, and so on. Nevertheless, there are also a series of emerging non-oncologic applications of radiomics, such as in assessing fibrosis of lung and liver.

To be more specific, the application of radiomics to study primary liver cancer and other liver diseases will help its early diagnosis and treatment and improve the disease survival and cure rate. Hence, it has broad application prospects. At present, relevant research is just emerging and developing rapidly, but still awaiting further maturity. In other words, present publications and literature give more questions than answers. Therefore, we summarize the progress of current research in this article, then point out the related deficiencies and the direction of future research.

## Hepatocellular carcinoma (HCC)

The radiomics research in liver disease is mainly focused on HCC [4–12] (Table 1). Radiomic features could be classified into different categories based on the approaches applied to calculate them, such as statistics-based, model-based, and transform-based (Table 2). The statistics-based features reflect the intensity distribution of the pixels or voxels within the ROI and they can be further classified into first-order, second-order, or high-order features, depending on whether they take spatial and topological information into consideration. In addition, features can be calculated not only on the base image, but also on images transformed using different filters, such as wavelet and Gabor filters. Those features belong to transform-based categories. So far, there are several available software programs to do radiomic analyses, which facilitate standardized settings for feature extraction. Representative open-source software packages include imaging biomarker explorer (IBEX), Chang Gung Image Texture analysis, and MaZda. Other software packages are commercial, like TexRAD^TM^ (Feedback plc, Cambridge, UK) and RADIOMICS^TM^ (OncoRadiomics, Maastricht, The Netherlands). It is worth mentioning that the features reported by current articles are often not standard and may have multiple meanings, which will influence the reproducibility and validation of the studies. Further research should report based on the reference manual of the image biomarker standardization initiative [13].

**Table 1.** Radiomics on hepatocellular carcinoma (HCC) in practice

Objective	Image type	No. of patients	Features	Results	Reference
Diagnosis	CT	80	Mean gray-level pixel intensity, entropy, standard deviation, kurtosis, and skewness in unfiltered images and 2-, 3-, 4-, 5-, and 6-mm filter, in total 30 features	The model, in distinguishing the lesion types (focal nodular hyperplasia, adenoma, or HCC) and normal liver, is up to 90%	[4]
Diagnosis	CT	109	Mean value of positive pixels, entropy	The model combining those two features discriminated the type of portal-vein thrombosis accurately, with an AUC of 0.99	[5]
Staging and grading	MRI	46	Mean intensity value, GLN	The low-grade HCCs have significantly larger mean intensity value and smaller GLN than high-grade HCCs (*P *<* *0.0005)	[6]
Therapeutic selection	CT	130	Wavelet-2-H, wavelet-2-V	Features were correlated with OS; patients can then be divided into groups to select the proper therapy	[7]
Therapeutic selection	CT	197	Gabor-1-90 (filter 0), wavelet-3-D (filter 1.0)	TACE group with higher Gabor-1-90 (>3.6190) or wavelet-3-D (>12.2620) may get better results if treated with sorafenib	[8]
Prognosis assessment	CT	127	–	8 and 15 features can predict DFS and OS, respectively	[9]
Prognosis assessment	PET	47	–	A scoring system is generated to predict PFS and OS	[10]
Prognosis assessment	CT	138	OS: compacity Local control: energy, gray-level non-uniformity for run	A single radiomics feature is significant to the OS of patients treated with volumetric modulated arc therapy and two are significant for local control	[11]
Surveillance	CT	215	21 radiomics features	A radiomics signature was built by selected features, which was significantly associated with early recurrence (*P *<* *0.001)	[12]

GLN, gray-level run-length non-uniformity; TACE, transcatheter arterial chemoembolization; OS, overall survival; DFS, disease-free survival; PFS, progression-free survival.

**Table 2.** The classification and meaning of commonly used and mentioned imaging features

Classification		Feature family	Description	Representative features (informal name)	Meaning
Statistics-based	First-order	Morphology	Describe geometric aspects of ROI	Volume	Counting of voxels in given volumes
				Compactness (compacity)	Measure for how sphere-like the volume is
				Sphericity	Also describe how sphere-like the volume is using a different algorithm
		Intensity-based statistics	Describe how gray levels are distributed within the ROI. Do not require discretization	Mean (mean intensity value)	The mean gray level of all the pixels within the ROI, including all the positive and negative gray levels. It reflects the average brightness of the ROI
				Mean value of positive pixels/ mean positive pixels	The mean gray level of all the pixels within the ROI that have a positive gray level
				Standard deviation	The SD of all the pixels within the ROI, which reflects the width of the distribution of intensities
				Kurtosis (median kurtosis)	The peakedness of the gray-level distribution within the ROI
				Skewness (median skewness)	A measure of asymmetry in the gray-level distribution within the ROI
				Energy	Calculated based on specific algorithm
		Intensity histogram	Describe how gray levels are distributed within the ROI by discretizing into gray-level bins	Entropy	An information-theoretic concept, discretize using a *fixed bin size* algorithm with 25 HU bins, extracted from a CT image, which reflects the irregularity of the ROI
	Second-order	Gray-level co-occurrence matrix	Describe how combinations of discretized gray levels of neighboring pixels or voxels are distributed along one of the image directions	Dissimilarity	Assess gray-level variations
	High-order	Gray-level run-length matrix	Assess run length, which is defined as the length of a consecutive sequence of pixels or voxels with the same gray level along one of the image directions	Gray-level run-length non-uniformity (gray-level non-uniformity for run)	To assess the distribution of runs over the gray values
Transform-based		Wavelets	Transformation using wavelet filter	Wavelet-2-H	A feature extracted after the transformation of images by wavelet
				Wavelet-2-V	A feature extracted after the transformation of images by wavelet
				Wavelet-3-D	A feature extracted after the transformation of images by wavelet
		Gabor	Transformation using Gabor filter	Gabor-1-90	A feature extracted after transformation by Gabor filter

ROI, region of interest.

### Diagnosis

In real clinical practice, the detection of HCC by visual analysis in most cases is not hard. However, some other hypervascular lesions in the liver may look like HCC and confuse the diagnosis of HCC, especially some benign lesions like focal nodular hyperplasia (FNH), hemangioma, and adenoma. Misclassification of HCC from those benign lesions will lead to failure to reach the best clinical decision, like unnecessary surgery or missing the best time for surgery.

Raman *et al.* [4] retrospectively evaluated the arterial-phase images of pathologically proven FNH, hepatic adenomas, HCC, and normal liver parenchyma with 17, 19, 25, and 19 cases, respectively, using computed tomography texture analysis (CTTA). Then they extracted 32 features (mean gray-level pixel intensity, entropy, standard deviation [SD], kurtosis, and skewness in unfiltered images, and 5 different filters, plus the size of the ROI and lesion enhancement) by Commercial CTTA software (TexRAD Ltd) and then construct a predictive classification model using the random-forest method with all of the 32 features. The accuracy of the model in distinguishing the lesion types and normal liver was up to 90% compared with two human readers with accuracies of 72.2% and 65.6%. In addition, pairwise comparison of each of the 32 features shows that 84% features in mean, SD, and entropy categories are statistically significant, whereas only 13% features in the kurtosis and skewness categories are associated with the lesion types. However, when comparing adenoma and HCC, none of the 32 features showed a significant difference. This raises a question: how does the random-forest model make the prediction between adenoma and HCC? Notably, the sample size is relatively small and the validation process of this research used a subset of the data used for building the model, which will result in higher accuracy than actuality and thus need further external validation. Future, with the aid of radiomics, it is possible to recognize benign liver lesions like FNH precisely and select the best treatment regimen.

Whether portal-vein thrombosis (PVT) is benign or malignant can determine the tumor stage and selection of treatment options, although it is easy to be misjudged by a radiologist’s subjective evaluation. The standard method of biopsy of PVT is invasive and may lead to false-negative results. In addition, it can cause complications like bleeding and metastasis, though with relatively low incidence. Canellas *et al.* [5] investigated 117 portal-venous-phase CT of 109 patients with 63 neoplastic and 54 benign thrombi using CTTA. The results showed that two features (mean value of positive pixels and entropy) discriminated the type of PVT accurately, with an area under the curve (AUC) of 0.99 for the model combining those two features. Although the differentiation by thrombus-density measuring with Hounsfield units (Hu) is also significant, the threshold values of Hu are determined by scanning protocols and the dose of contrast media. Comparing radiomics features selected by models to that, radiomics is more constant and reliable.

### Staging and grading

The staging and grading of HCC are vital to decide the optimal treatment and prognosis. Theoretically, that depends on the histopathological features of the tumor tissues after surgical resection. Fine-needle biopsy is a common way to assess HCC malignancy preoperatively. However, it is not fully desirable in daily clinical practice due to some occasional complications like bleeding and metastasis, though not very common. On the contrary, non-invasive radiomics analysis can detect the whole tumor tissue and surrounding parenchyma, which makes a potential way to evaluate biological aggressiveness in the future.

In order to find the correlation between the features and histological grading, Zhou *et al.* [6] utilized the method of texture analysis to extract the features of Gd-DTPA contrast-enhanced MRI images from 46 consecutive patients with resected HCC. The result showed that two features called the mean intensity value and gray-level run-length non-uniformity (GLN) had better performances in arterial-phase images. Specifically, the low-grade HCCs (Edmondson grades I and II) have significantly larger mean intensity values and smaller GLN than high-grade HCCs (Edmondson grades III and IV) (*P *<* *0.0005).

### Therapeutic selection and prognosis assessment

Li *et al.* [7] investigated 130 patients with single HCC (>5* *cm) in Barcelona Clinic Liver Cancer (BCLC) stage B or C who underwent either liver resection (LR) or transcatheter arterial chemoembolization (TACE) by CTTA. They found that the parameter Wavelet-2-H in LR patients and wavelet-2-V in TACE were correlated with overall survival (OS). Then, based on that result, patients were divided into four groups (LR+, LR–, TACE+, TACE–) according to the median of the parameter. Further, they estimated whether, if LR+ patients were treated with TACE, they would have severe compromises in OS. In contrast, TACE– patients would get better therapeutic outcomes by undergoing LR and similarly TACE is beneficial to LR– and TACE+ patients. Thus, such radiomic parameters have the potential to help in selecting the correct therapeutic plans for each individual.

Other research has focused on whether HCC patients who have undergone TACE should receive sorafenib simultaneously in order to control the level of vascular endothelial growth factor (VEGF) [8]. Radiomic features from 197 patients who had received TACE therapy were extracted. The results showed that Gabor-1–90 (filter 0) and wavelet-3-D (filter 1.0) were highly correlated with time to progression (TTP) and OS, respectively. The TACE group with higher Gabor-1–90 (>3.6190) or wavelet-3-D (>12.2620) had shorter TTP or OS compared with the other patients in the TACE group and TACE plus sorafenib group (*n *=* *64), even though the baseline characteristics between these groups are comparable. So we have reason to believe that such subgroups may have a better prognosis if they are treated with TACE plus sorafenib.

Moreover, Akai *et al.* [9] used a random forest that selected 8 and 15 texture features from a total of 96 features to predict the disease-free survival (DFS) and OS, respectively, based on 127 patients who underwent LR for HCC. The model trained on these features and divided the patients into two groups—high and low predicted individual risk. A multivariate Cox proportional-hazards model showed that a high predicted individual risk was an independently bad prognostic factor. Henceforth, patients with resectable HCC can be assessed preoperatively by such factors to decide whether they should undergo surgery directly or receive other adjuvant treatments first.

Similarly, the prognosis for unresectable HCC patients who have received non-surgical treatment can also be predicted by radiomics analysis. Blanc-Durand *et al.* [10] retrospectively analysed the pretreatment ^18^F-FDG PET of 47 patients assigned to transarterial radioembolization using Yttrium-90 (^90^Y-TARE) from the features extracted by texture and intensity analyses of the entire liver instead of the ROI. They generated a scoring system (pPET-RadScores) using the method of LASSO Cox regression. The cut-off value of this score system can divide the patients into high- or low-risk groups, which is significantly correlated with progression-free survival (PFS) and OS (both *P *<* *0.01). In addition, together with the BCLC staging system and alpha-fetoprotein (AFP) level, pPET-RadScores is confirmed to be an independent predictor for PFS and OS.

In another study, Cozzi *et al.* [11] identified a single radiomics feature of ‘compacity’, which is significant to OS of patients treated with volumetric modulated arc therapy, by analysing the ROI in non-contrast-enhanced CT images (*P *<* *0.00001, AUC* *=* *0.8014). And two other features, namely ‘energy’ and ‘gray-level non-uniformity for run’, are significant to local control, although the AUC is only around 0.6. Another group of patients are undergoing a validation study.

### Surveillance

Undoubtedly, partial hepatectomy is the optimal choice to cure most cases of HCC. However, with the 5-year recurrence rate reaching ∼50% [14], a large number of post-operative patients still suffer from the disease and recurrence becomes the main cause of their death. Previous research has revealed that HCC patients with recurrence after <1 year would have a poorer prognosis than those with late recurrence (>1 year) [15]. Thus, if we can detect the patients who have a high possibility of suffering from early recurrence, then it is possible for us to intervene by a closer follow-up schedule and post-operative adjuvant therapy, aiming to sustain a longer survival after surgical resection.

Zhou *et al.* [12] constructed a model based on 21 radiomic features chosen from 300 candidates that are significantly associated with early recurrence (*P *<* *0.001), with AUC, sensitivity, and specificity of 0.817, 0.794, and 0.699, respectively. The result is better than the model based on clinical features (including age, gender, HBsAg, HCV-Ab, AFP level, ALT, γ-GGT, AST, Child–Pugh grade, BCLC stage, and history of preoperative adjunctive treatment). The model combined with radiomics and clinical features had even better results, with AUC, sensitivity, and specificity of 0.836, 0.824, and 0.7082, so it may become a potential powerful tool for stratifying patients on recurrence risk preoperatively.

## Non-HCC malignant lesions

Likewise, it should also be a promising field to investigate other kinds of malignant lesions like cholangiocarcinoma, liver sarcoma, and liver metastases by means of radiomics. But, maybe because of the restraint of relatively lower morbidity, the amount of research is very limited.

Some imaging features summarized by the experience of radiologists have validated clinical significance. Aherne *et al.* [16] recruited 66 patients with surgically resected intra-hepatic cholangiocarcinoma and were eager to find the associations between preoperative CT-imaging features and OS. They found that three features (necrosis, satellite nodules, and vascular encasement) were significantly associated with OS. Although the three features described are qualitative findings recognized by radiologists, they inspire a way to explain the biological implications of radiomic features. In other words, if we can replicate the research above by extracting radiomic features and try to find the connection between radiomic and qualitative features, we may provide a more easily understand explanation of the potential clinical meaning of radiomic features.

Liver metastasis is another important type of liver lesion. Lubner *et al.* [17] performed CTTA on pretreatment contrast-enhanced CT from 77 patients with single liver metastasis, finding that mean positive pixels, entropy, and SD are significantly related to tumor grade and entropy is also predictable for OS. They also compared the results of texture analysis using 2D pixels from a single slice with the results using 3D voxels from multiple slices, though there was no significant differences. Reimer *et al.* [18] tried to assess the therapy response to TARE for liver metastases through radiomic analysis of post-treatment MRI images. They revealed that median kurtosis in arterial-phase MRI and median skewness and kurtosis in the venous phase could significantly discriminate patients on whether they have a progressive disease. And, compared to response-evaluation criteria in solid tumors 1.1 (RECIST 1.1), those radiomics features could predict the therapy response even earlier. Future research should focus on whether radiomics can tell the differences between liver metastasis and primary liver cancer. Besides, are there any possibilities of detecting the origin of liver metastasis by means of radiomics?

## Benign liver diseases

### Non-alcoholic fatty liver disease (NAFLD)

Since radiomics can not only detect on extremely subtle regional change, but also analyse the average situation of the whole organ or tissue, it is appropriate for radiomics to evaluate the range and severity of diffuse lesions like liver steatosis. NAFLD is the most common liver disease in developed countries. Taking the USA as an example, the results of the US National Health and Nutrition Survey showed that the prevalence of NAFLD increased from 5.5% during 2005–2008 to 11% during 1988–1994, holding the proportion of 75% in chronic liver disease from 47% [19]. The early diagnosis and intervention of NAFLD are the most important tasks in the field of liver disease in the future.

The natural course of NAFLD patients may progress from non-alcoholic fatty liver (NAFL) to non-alcoholic steatohepatitis (NASH), which leads to liver fibrosis and eventually progresses to cirrhosis. Patients with NAFL have a lower risk of fibrosis than NASH patients [20]. Besides, NAFLD has been recently identified as a risk factor for HCC. Patients with NASH has an estimated HCC incidence of 1.6% over 15 years [21]. So early recognition of NASH is critical to delaying the progression of NAFLD. However, there is currently no imaging method routinely used to identify NAFL and NASH, and liver biopsy is needed for diagnosis [22]. Radiomics provides the possibility of non-invasive classification of NAFLD.

Naganawa *et al.* [23] first applied texture analysis on non-contrast-enhanced CT to detect NASH. A total of 88 patients suspected of NASH based on abnormal liver function were divided into the learning data set (*n *=* *53) and the validation data set (*n *=* *35), and then subdivided by the level of serum hyaluronic acid (cut-off, 50 μg/L), since it can predict fibrosis, which would affect the results of texture analysis. The reference diagnosis of patients with or without NASH was confirmed by liver biopsy. For patients without suspicion of fibrosis, the NASH prediction model was based on features of mean (without filter) and skewness (2-mm filter) with an AUC of 0.94 for the validation data set. In contrast, the model for patients with suspicion of fibrosis was not satisfying, with an AUC of only 0.60. One of the limitations of this study is that the sample only included patients with a suspicion of NASH, which caused the pretest possibility of NASH to be higher than the normal population and may lead to better results than in reality. Besides, for patients with pre-existing liver fibrosis, since the predictive model did not perform well, an alternative test should be sought for them.

Research has suggested that MRI, compared with transient elastography and CT, is a more promising technique to accurately diagnose NAFLD [24]. Especially, calculating the proton density of the fat fraction based on MRI (MRI-PDFF) has the potential to replace liver biopsy as the gold standard for the diagnosis and grading of NAFLD [25]. Future studies should use MRI as the source of information.

### Liver fibrosis

The application of texture analysis to CT images can also be used to analyse the extent of liver fibrosis [26, 27]. Radiomics can be applied not only to conventional imaging examinations such as CT and MRI, but also to those specific tests in the liver-disease field like 2D shear-wave elastography (2D-SWE), which may have the potential to improve the accuracy of the diagnosis of liver fibrosis.

Although 2D-SWE has been used widely to assess the stiffness degree of the liver, different hospitals have utilized different cut-off values for diagnosing liver fibrosis, which has made it unfeasible to compare among different institutions. Wang *et al.* [28] conducted a prospective study to use the method of deep-learning radiomics to analyse 2D-SWE, including 398 patients with chronic hepatitis B with 1,990 images from 12 hospitals. The AUCs of the model created to predict different stages of liver fibrosis were 0.97 for cirrhosis (F4), 0.98 for ≥F3, and 0.85 for ≥F2, which were significantly superior to 2D-SWE (except for ≥F2), AST-to-platelet ratio index, and fibrosis index based on four factors. It also showed that the inclusion of more images would improve the diagnostic accuracy of the model. So, we have reason to believe that the model will achieve a better diagnostic efficacy with the gradual training of the model as more information from new patients is added to the data set.

Since the result of the SWE will be affected when facing the situation of ascites, obesity, and steatosis, Li *et al.* [29] combined different modalities of ultrasonography, including original radiofrequency, contrast-enhanced micro-flow, and conventional features, in order to improve the accuracy of discriminating significant fibrosis (≥F2). They also compare the performance of different machine-learning algorithms. Although the result was not improved, similar ideas can be applied in future research.

### Portal hypertension

Portal-vein hypertension, mainly caused by liver cirrhosis, is related to esophagogastric varices and hypersplenism, which may lead to a bad clinical outcome. The measurement of the pressure of the portal vein, however, is invasive and thus cannot be easily accepted by routine patients in spite of minimal side effects [30]. Liu *et al.* [31] tried to detect for clinically significant portal hypertension, which is defined as a hepatic venous pressure gradient >10 mmHg, by contrast-enhanced CT using the method of radiomics. The performance of this model was validated by external cohorts, with a C-index ≥0.800 (95% confidence interval: 0.614–0.986). The result of this study supports the potentiality of the non-invasive measurement of portal pressure in the future.

## Liver disease and radiogenomics

The biological behavior of a tumor is closely related to its gene-expression profile. Biopsy and tumor resection are the two major existing methods to assess gene expression with certain accuracy. The shortcomings of biopsy, obviously, are hemorrhage and tumor metastasis, which hinder its routine practice in spite of a very low incidence. In addition, genetic evaluation before surgical intervention is needed for appropriate individualized management. Therefore, preoperative and non-invasive examination of tumor-gene expression is a clinically ideal target. A study by Pinker *et al.* [32] showed that 78% of HCC gene-expression profiles could be reconstructed with 28 image features and radiogenomics—the method of judging gene expression by radiomics [33]—has potential clinical advantages.

### Hepatocellular carcinoma

Microvascular invasion (MVI) has been identified as a powerful independent predictor for early recurrence and poor prognosis after surgical resection of HCC [34]. But the diagnosis of MVI relies on histopathological testing after surgery. Thus, for clinical decision-making on HCC patients, a non-invasive test that can detect the presence of MVI is important. Traditional imaging cannot reveal MVI because of the poor resolution. Moreover, the results for predicting MVI with the traditional imaging trait of a ‘non-smooth tumor margin’ in preoperative CT imaging are unsatisfactory, with a sensitivity of 66% and specificity of 86.5% [35].

Chen *et al.* [36] isolated 91 gene-expression profiles associated with MVI in HCC. Based on this conclusion and the method of radiogenomics, Banerjee *et al.* [37] isolated a cluster of radiomic features that were correlated with MVI gene-expression profiles and then established a model called RVI (Radiogenomic venous invasion) for predicting MVI. Its sensitivity, specificity, and accuracy can reach 89%, 76%, and 94%, respectively, which are better than the results when using traditional imaging traits. They also found that RVI is associated with HCC early recurrence and poor prognosis. Renzulli *et al.* [38] found that the combination of non-smooth tumor margins, peritumoral enhancement, and the radiogenomic features could rule out the effect of tumor size on predicting MVI and was an independent predictor with a positive predictive value of 0.95.

### Liver metastasis

Radiogenomics can be used to determine the KRAS-mutation status of colorectal cancer liver metastasis (CRLM) and thereby the prognosis of surgical resection. A study by Margonis *et al.* [39] showed that, in CRLM patients with KRAS mutations, surgical resection reduced the risk of recurrence and prolonged DFS, whereas patients with KRAS wild-type tumor did not benefit from surgical resection. Ji *et al.* [40] proposed that radiogenomics could be used to predict the KRAS mutation of CRLM preoperatively. At present, radiogenomic studies for predicting the KRAS-mutation status of solid tumors are still limited.

## Perspective

Essentially, the significance of radiomics is to dig deeper for information on traditional medical imaging to make up for the shortcomings of the human eye. Therefore, radiomics should no longer be dedicated to what can already be achieved by radiologists, such as the diagnosis of liver cancer and fatty liver. Conversely, topics such as the assessment of disease severity, selection of therapeutic options, and prognosis prediction, which are impossible to achieve by traditional radiology, should be the focus of future radiomic research. As for liver disease, the prognostic prediction of hepatic malignancies including, but not limited to, HCC, the accurate discrimination of NASH from NAFL, and the evaluation of its severity are promising aspects that need to be further studied.

At present, research on radiomics is still in its infancy and there are no standardized and unified standards for the complicated research process. For the selection of ROI, there is currently no suitable algorithm to calibrate tumor regions. Most studies calibrated ROI by radiologists, which increases the amount of pre-work, while calibration by different people will have an impact on the subsequent establishment of the model, leading to limited reproducibility of the results and comparability between studies [41]. Also, a lack of standardization in reporting the results of research often makes it confusing for readers. For example, some of the features mentioned above are not declared fully and formally, such as there are different feature ‘means’ in both intensity-based statistical features and intensity histogram features with different ways of calculation. We propose that future studies should report features based on the ‘Image biomarker standardisation initiative’ using formal nomenclature and corner marks. Furthermore, traditional machine-learning algorithms such as random forests and deep-learning algorithms like the neural network that have emerged in recent years can both be used for the establishment of radiomics models. The algorithms used by each type of research are different. Still, there is no research to prove which algorithms are the most suitable for such work. Finally, most of the current research results are still in the training sample stage, so the high accuracy of the model does not reflect its actual predictive ability. Whether the model is really effective or not depends on the validation phase by the test sample.

In conclusion, while initial studies looking at radiomics have been very promising, there has been poor standardization and generalization of radiomic results, which limit the translation of this approach into clinical practice. Clear limitations of this field are emerging, especially with regard to data-quality control, repeatability, reproducibility, generalizability of results, and issues related to model overfitting. To address those problems, we propose that future radiomic research should be assessed via the radiomics quality score established by Lambin *et al.* [3]. By doing so, radiomics studies can be more comparable and increase its potential to be applied in future clinical practice. Foreseeably, the advance in radiomics will largely contribute to the development of personalization and precision medicine.

## Authors’ contributions

W.H. searched the papers and wrote the article. H.Y. and H.X. revised it. Y.M. designed the ideas of the article and approved publishment.

## Funding

None.

## Conflicts of interest

None declared.
